# Identification of a de novo splicing mutation in the *CSF1R* gene in a Chinese patient with hereditary diffuse leukoencephalopathy with spheroids

**DOI:** 10.1007/s10072-021-05755-5

**Published:** 2021-11-18

**Authors:** Xinwei Wu, Congcong Sun, Xingbang Wang, Ying Liu, Wei Wu, Guoyong Jia

**Affiliations:** 1 Department of Geriatrics, Qilu Hospital, Cheeloo College of Medicine, Shandong University, Shan Dong, Jinan, 250012 China; 2 Department of Neurology, Qilu Hospital, Cheeloo College of Medicine, Shandong University, Shan Dong, Jinan, 250012 China

**Keywords:** Hereditary diffuse leukoencephalopathy with spheroids, CSF1R, De novo, Splicing mutation

## Abstract

**Objective:**

To report a de novo splicing mutation in the *CSF1R* gene in a patient with hereditary diffuse leukoencephalopathy with spheroids (HDLS).

**Methods:**

A 42-year-old Chinese woman with constant weakness on her left lower extremity was recruited in the current study. Detail medical history and clinical characteristics were reviewed. Brain magnetic resonance imaging (MRI), whole-exome sequencing, and Sanger sequencing were performed with bioinformatics analysis.

**Results:**

The Chinese HDLS patient with no HDLS family history exhibited a de novo splicing mutation (c.1754-10 T > A) in the *CSF1R* gene. This mutation was located at the splice site of intron 12 and resulted in the skipping of exon 13 from the *CSF1R* mRNA. This finding constitutes the first de novo splicing mutation ever reported in HDLS. Furthermore, MRI abnormalities had been reported at least 6 months prior to the onset of the patient’s clinical phenotype.

**Conclusion:**

Our study indicates that the diagnosis of HDLS should be considered even in the absence of a family history and can help deepen the clinical and genetic understanding of HDLS.

## Introduction

Hereditary diffuse leukoencephalopathy with spheroids (HDLS) is a rare autosomal dominant inherited disease first described by Axelsson R et al. in a Swedish family in 1984 [1]. It is a neurodegenerative disease characterized by the adult onset of progressive cognitive impairment, stroke-like motor disorders, behavioral and emotional changes, sensory disturbance, epilepsy, and dizziness, among others [2, 3]. Brain magnetic resonance imaging (MRI) of patients with HDLS reveals asymmetric leukoencephalopathy, with the main involvement of frontoparietal deep white matter, as well as brain atrophy [3, 4]. Neuropathological features included degeneration of cerebral white matter (WM) and atrophy of the corpus callosum [5]. Histopathologic studies demonstrated loss of myelinated fibers and abundant axonal spheroids mainly containing aggregates of intermediate filaments or organelles that were predominantly vesicular and lamellar [6].

The diagnosis of HDLS is challenging owing to its significantly variable phenotypes [7]. It is easily misdiagnosed with other leukoencephalopathy, small vessel diseases, and neurodegenerative diseases such as frontotemporal dementia (FTD). In 2012, mutations in the colony-stimulating factor 1 receptor (*CSF1R*) gene, located on chromosome 5 (*5q32*), were identified as the cause of HDLS [8]. The CSF1R is a cell surface receptor which is crucial in development and innate immunity in the central nervous system [9]. In this study, we report a de novo splicing mutation (c.1754-10 T > A) in the *CSF1R* gene in a HDLS Chinese patient with no family history of the disease. The mutation was located at the splice site of intron 12, which resulted in the skipping of exon 13 from the CSF1R mRNA. Although more than 60 loci mutations in the *CSF1R* gene have been linked to HDLS [10, 11], de novo mutations were rarely reported [7, 12, 13]. Besides, we reported the MRI changes in the different stages of the patient and found that diffusion-weighted imaging (DWI) revealed abnormal hyperintense signals even in the presymptomatic stage of HDLS.

In this study, we report detailed clinical imaging features of a Chinese HDLS patient with no HDLS family history who exhibited a de novo splicing mutation (c.1754-10 T > A) in the *CSF1R* gene. Overall, our study can help deepen the clinical and genetic understanding of HDLS.

## Materials and methods

### Patient history and clinical data

A 42-year-old Chinese woman with no medical history complained of a sudden onset of constant weakness on her left lower extremity for 2 months in October 2019. About 1 month later, her left upper limb also became weak. The patient was born at term to non-consanguineous parents following a normal pregnancy and delivery. At the age of 23, the patient gave birth to a healthy baby girl. The patient was admitted to the local hospital and administered platelet aggregation inhibitor, statins, and other drugs. Her condition gradually improved and she could walk normally when she came to our hospital for further examination.

### Physical examination

At the time of admission to our hospital, the patient’s blood pressure was 122/71 mmHg, and her heart rate was 75 beats per minute. She had no significant deficits in memory, directive force, and learning or calculation abilities. Furthermore, no obvious deficits were observed in the cranial nerve examinations, and the muscle force of all the limbs was level 5. The muscular tension of her left limbs increased, which led to positive Babinski and Chaddock signs. Her mini-mental state examination (MMSE) score was 24 points, and her Montreal Cognitive Assessment (MoCA) score was 22 points.

### DNA extraction

With the informed consent of the patient, 2 mL of blood was collected, and DNA was extracted by a genomic DNA extraction kit (Quan-Jin Biotech Co., Ltd., Beijing, China). Finally, DNA purity and integrity were assessed using spectrophotometry (Thermo Fisher Scientific, Waltham, MA, USA) and agarose gel electrophoresis, respectively.

### Whole-exome sequencing (WES) and data analysis

WES was performed by Annoroad Genomics Co., Ltd. (Beijing, China) and the methods were based on previous studies [14, 15]. The AgilentV6 exon targeting sequence enrichment system was used to capture the whole-exome sequences, and the enriched sequences were double-ended sequenced by Hiseq sequencing platform (PE150). The coverage of the target area was more than 99%, and the average depth of the target area was higher than 20 × , accounting for 98% of all sequencing depths. The original sequencing data of the patients were mapped to the human reference genome UCSC HG19 through the comparison software BWA, and the comparison result file in BAM format was obtained. The mutation analysis software GATK was used to analyze all the mutation sites, and Annoroad software was used to make functional annotations for all the mutation sites.

### Validation by Sanger sequencing

Sanger sequencing was performed in the protestors to validate identified pathogenic variants using whole-exome sequencing. CSF1R-F: GCTG CCCT GTCA CTGT GTA and CSF1R-R: TCGT TTCC CATC CCAG GA are based on the reference genome sequence of the human genome from NCBI GenBank by Primer 3 designed for the candidate locus and PCR under the following conditions: 95 °C/4-min initial denaturation, followed by 95 °C/30-s denaturation 30 cycles, 52 °C/30-s annealing, 72 °C/45-s extension, and 72 °C/5-min extension.

### RT-PCR analysis

Total RNA was extracted from the peripheral blood of the patient and a healthy control. Reverse transcription of RNA into cDNA was performed using the TaKaRa RNA PCR Kit according to the manufacturer’s protocol. The cDNA was amplified with primers (F: CCGG ATGA GTTC CTCT TCAC, R: CATG ATCT TCAG CTCG GACA) spanning from exons 11 to 14. We then separated the RT-PCR products using 2% agarose gel. Sanger sequencing was also performed on the cDNA.

## Results

### MRI scan

**Fig. 1:**
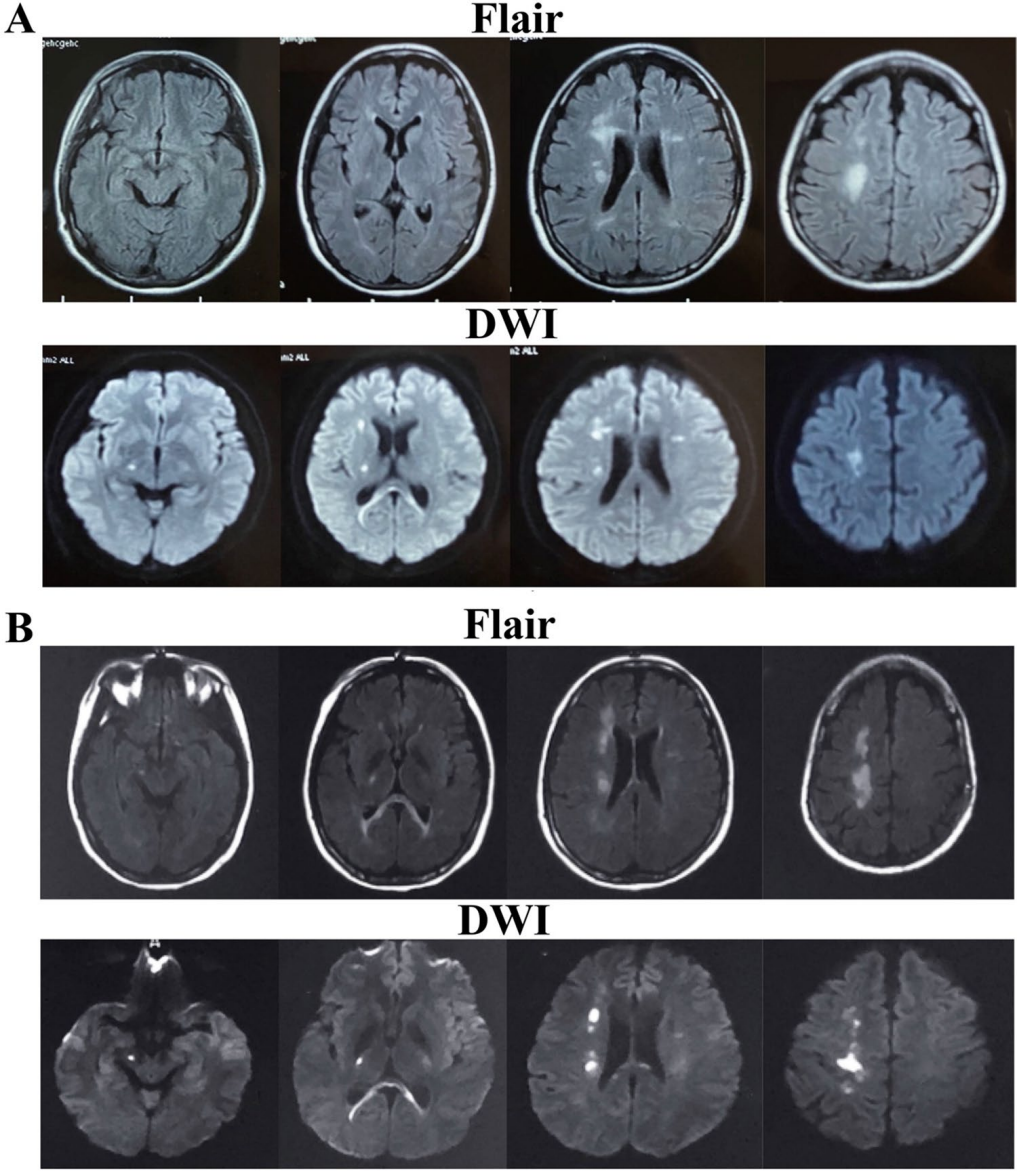
Magnetic resonance imaging (MRI) of the patient. **A** In March 2019, when the proband had no symptoms, the fluid-attenuated inversion recovery (FLAIR) and diffusion-weighted imaging (DWI) hyperintense signals were observed in the bilateral corona radiata, the splenium of corpus callosum, and the right basal ganglia region. **B** In October 2019, when the proband complained of the weakness of her left lower limb, MRI demonstrated more abnormal signals in the right corona radiata regions and the centrum semiovale than those in March 2019

During a routine checkup in March 2019, DWI hyperintense signals were observed in the bilateral corona radiata, the splenium of the corpus callosum, the centrum semiovale, and the right basal ganglia region (Fig. 1A). The patient did not take these signs seriously and refused further examinations as she had no neurological symptoms. In October 2019, when she complained of the weakness of her left lower limb, brain MRI revealed multiple patchy high T1 and T2 signals in the splenium of the corpus callosum, the bilateral corona radiata regions (more significantly on the right side), the centrum semiovale, and the right basal ganglia region. T2 fluid-attenuated inversion recovery (FLAIR) and DWI revealed more hyperintense signals in the right corona radiata regions and the centrum semiovale than those in March 2019. Finally, contrast-enhanced MRI scan revealed no obvious abnormal intensification (Fig. 1B), and magnetic resonance angiography did not indicate vascular stenosis in the brain.

### Laboratory findings and cerebrospinal fluid (CSF) test

A blood routine test of the patient revealed a hemoglobin (Hb) concentration of 94 g/L, a hematocrit of 31.2%, a mean corpuscular volume of 78.4 fL, and an erythrocyte sedimentation rate of 42 mm/h. Other blood analyses, including hepatorenal function, C-reactive protein, vitamin B12, TORCH screening, tumor makers, anti-neutrophil cytoplasmic (ANCA) antibody, anti-extractable nuclear (ENA) antibody, thyroid function, adrenocorticotropic hormone, syphilis serology, and HIV test, were all normal/negative. The CSF pressure was 140 mmH_2_O. Moreover, the number of lymphocytes, the protein level, and the biochemical tests of the CSF were in the normal range. Finally, specific antibodies (AQP4/MOG/MBP) did not detect any signs of demyelinating diseases in the central nervous system (CNS).

### Genetic tests

**Fig. 2:**
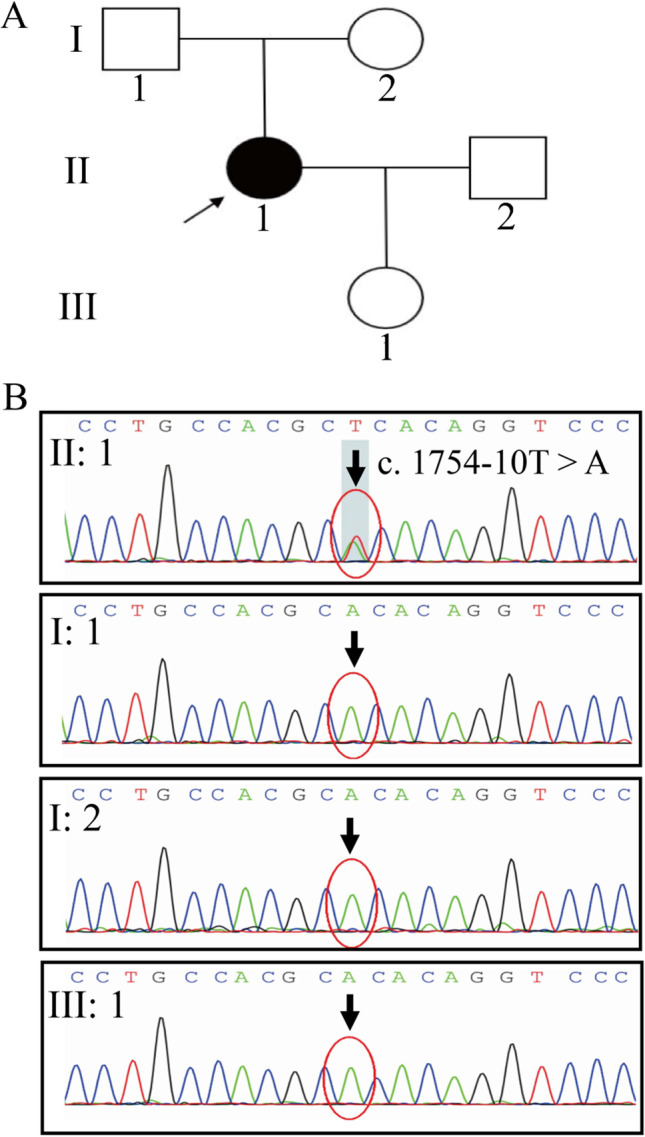
**A** Pedigrees of the family. The proband (patient II-1) is indicated by the arrow. **B** Sanger sequencing of the patient, her parents, and her daughter. The proband exhibited a de novo splicing mutation (c.1754-10 T > A) in intron 12 of *CSF1R* gene

**Fig. 3:**
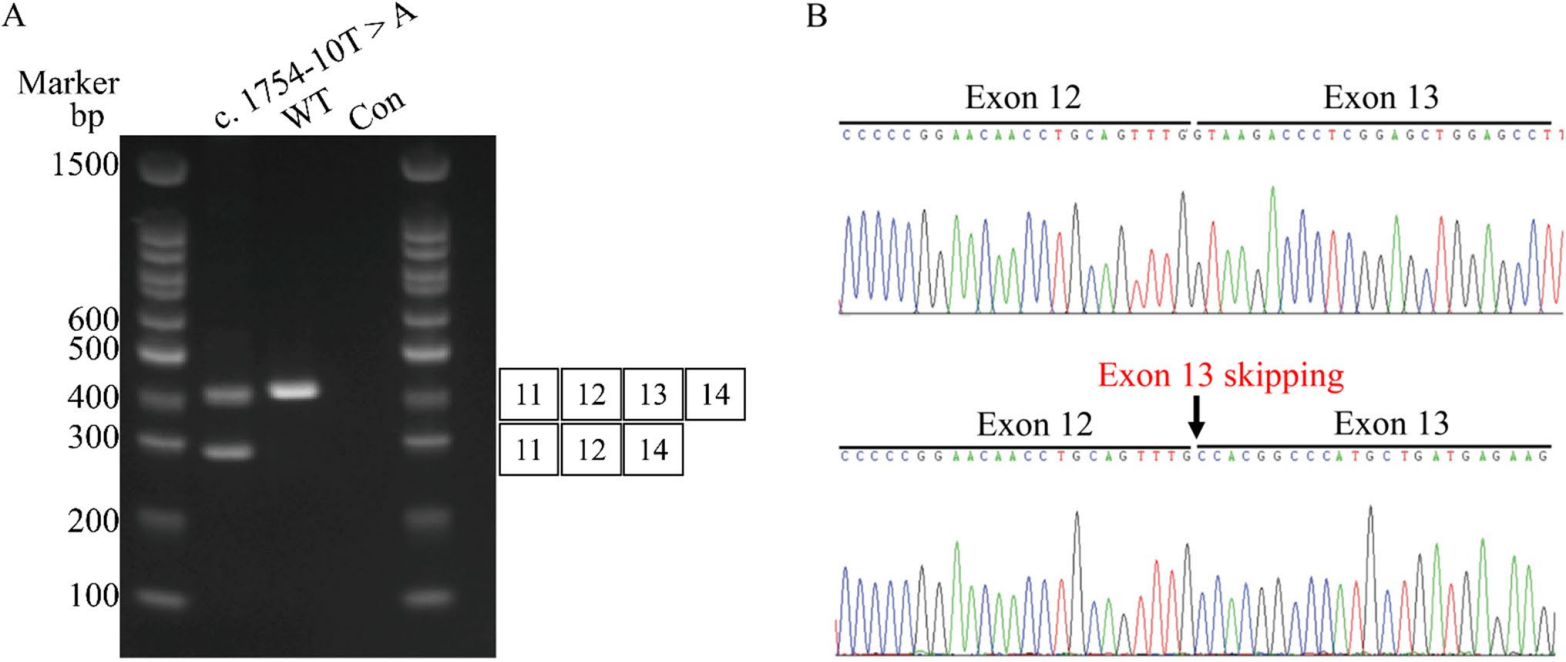
**A** Agarose gel showing the RT-PCR results of c.1754-10 T > A mutation in the proband, wild-type (WT) *CSF1R*, and control. **B** Sanger sequencing confirmed the c.1754-10 T > A mutation on the *CSF1R* gene caused exon 13 (105 bp) skipping during mRNA splicing

To identify the presence of inherited diseases, we extracted genomic DNA from the peripheral blood of the patient and performed WES. WES revealed a c.1754-10 T > A mutation in the *CSF1R* gene (Fig. 2A II-1). The presence of this mutation was confirmed by Sanger sequencing (Fig. 2B). The mutation was not observed in other family members (the patient’s parents and daughter) who exhibited no neurological symptoms. The mutation was absent in the Exome Aggregation Consortium database, the 1000 Genomes Project, and the Human Gene Mutation Database. The variant was highly conserved in different species (as observed using the MutationTaster software). Both the SIFT and PolyPhen-2 software predicted that this mutation was deleterious on the CSF1R protein structure and function. Subsequently, we extracted the total RNA from the peripheral blood of the patient and from a healthy control to determine the influence of the mutation on mRNA splicing. RNA was reverse transcript into cDNA, and then, cDNA was amplified with primers spanning from exons 11 to 14. Agarose gel showed only one 459-bp band in the sample of the healthy control and two bands in the patient’s sample, one with 459 bp and another smaller than 459 bp (Fig. 3A). This suggests that the mutation produced a truncated, alternatively spliced isoform. Sanger sequencing was also performed on the cDNA, confirming that the c.1754-10 T > A mutation on the *CSF1R* gene caused exon 13 skipping during mRNA splicing (Fig. 3B).

### Diagnosis and prognosis

**Fig. 4:**
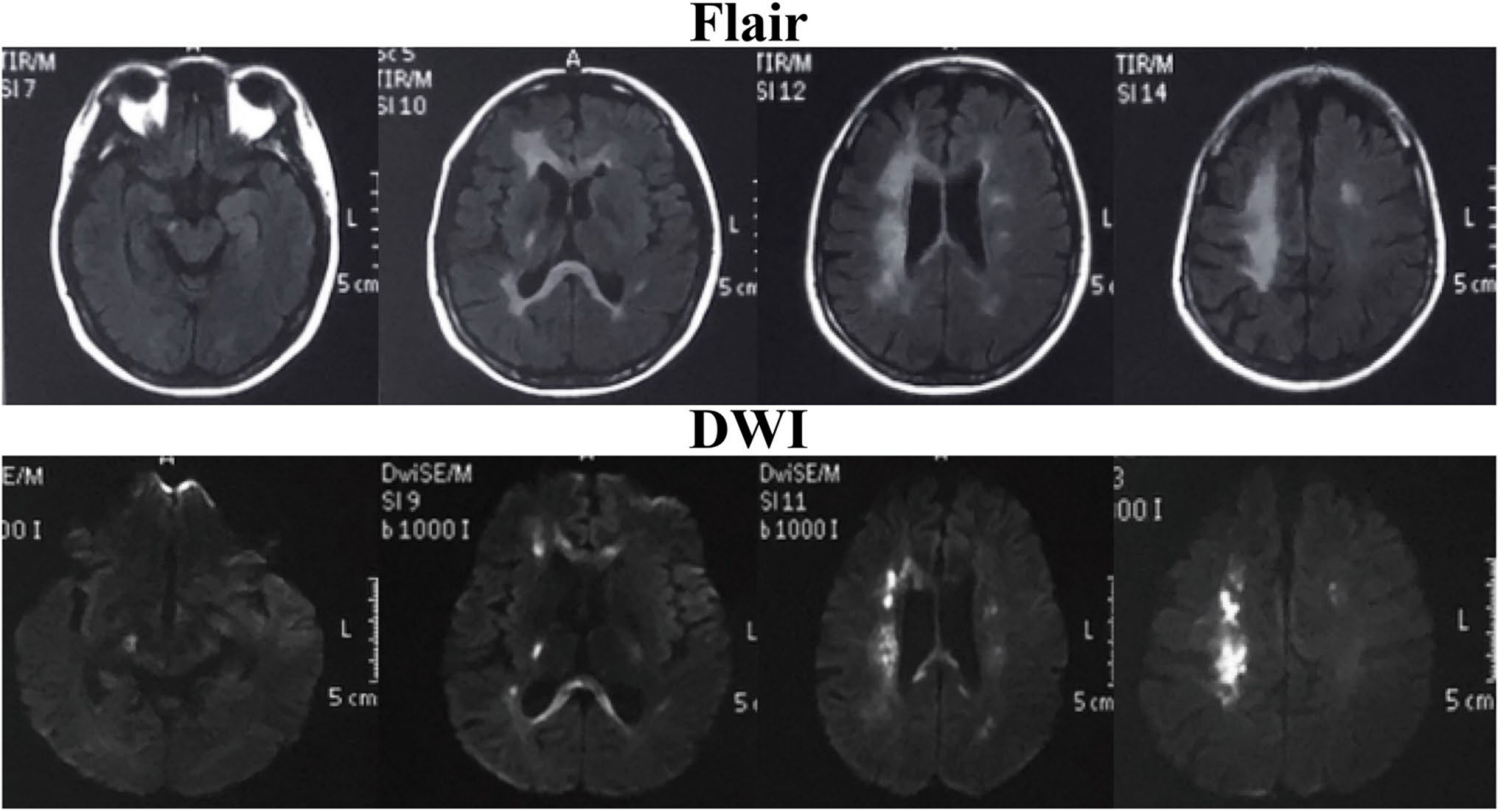
More abnormal signals in the splenium of the corpus callosum, bilateral corona radiata regions, centrum semiovale, and right corticospinal tract were detected in October 2020 when the proband suffered from recurrent weakness of the left limbs

At first, the patient was diagnosed with acute ischemic stroke and treated with aspirin enteric-coated tablets (100 mg/day), clopidogrel (75 mg/day), and atorvastatin (20 mg/day). After a series of routine examinations, we considered that she suffered from “acute ischemic stroke due to unknown reasons,” possible “primary angiitis of the central nervous system (PACNS),” and “iron deficiency anemia.” She was then given diagnosis therapy consisting of a glucocorticoid and an immunosuppressor. Then, the genetic test revealed the mutation in the *CSF1R* gene. Based on the clinical symptoms, MRI characteristics, and genetic test, the proband was diagnosed with HDLS. One year later in October 2020, the patient suffered from recurrent weakness of the left limbs. At that time, brain MRI revealed more abnormal signals in the splenium and rostrum of the corpus callosum, bilateral corona radiata regions, centrum semiovale, and right corticospinal tract, which significantly progressed than in 2019 (Fig. 4).

## Discussion

Here, we reported a de novo splicing mutation (c.1754-10 T > A) in the *CSF1R* gene in a Chinese HDLS patient with no family history of the disease. The age of the disease onset was 42 years old, which is consistent with the previous literature. HDLS often occurs in the age range of 40–50 years, but this has also been found to occur in the age range of 17–78 years [16, 17]. The pathological basis of HDLS is diffuse leukoencephalopathy with spheroids or demyelination [18]. Prominent clinical symptoms related with leukoencephalopathy include cognitive impairment as well as emotion and motor disorders, among others [19, 20]. In this case report, a female patient experienced stroke-like manifestations, and her hemiparesis was associated with an abnormality in the right corticospinal tract.

Like most HDLS patients, this patient went through a series of medical complications before being diagnosed, suggesting that HDLS should be differentiated from a number of diseases, especially other types of leukodystrophies, such as autosomal dominant arteriopathy with subcortical infarcts and leukoencephalopathy (CADASIL), Alexander disease, X-linked adrenoleukodystrophy, and metachromatic leukodystrophy [7, 21]. CADASIL usually manifests itself through migraines, stroke-like episodes, and dementia, and the involvement of the temporal tip could be considered pathognomonic [22]. In the case of Alexander disease, MRI usually reveals that the brainstem, specifically the medulla region, is involved [23]. Conversely, enzymatic tests are helpful for the diagnosis of X-linked adrenoleukodystrophy and metachromatic leukodystrophy [24]. The case described here should also be differentiated from acute ischemic stroke. In HDLS, continuous hyperintense signals are observed in DWI, and in acute ischemic stroke, these signals are only detected in an early stage of the disease.

Brain MRI of HDLS patients is characterized by high, bilateral, symmetrical, or asymmetrical white matter T2 signals. The frontotemporal lobes, corpus callosum, and U-fibers are typically affected [25], whereas the involvement of the thalamus and basal ganglia is rarely reported [3]. Furthermore, DWI demonstrates a restricted diffusion of these lesions. Notably, in the case reported here, MRI abnormalities have been observed at least 6 months prior to the onset of the clinical symptoms, which indicates that the typical imaging changes in HDLS preceded the onset of the clinical phenotype. Our finding is in accordance with that of Van Gerpen et al. [26], who reported an asymptomatic imaging study with frontal white matter abnormalities detected 5 years before the onset of the clinical symptoms.

In 2011, Rademakers et al. [12] confirmed that a mutation in the *CSF1R* gene was the genetic basis of HDLS. The *CSF1R* gene includes 24 exons and encodes for the CSF1R protein, which contains 972 amino acid residues [27]. Until now, more than 60 *CSF1R* mutations, including missense mutations, point mutations, frameshift mutations, and splicing mutations, have been identified as the genetic cause of HDLS [28]. HDLS is an autosomal dominant disease, but the reported sporadic cases [7, 12, 13] suggested that the diagnosis of HDLS should be suspected even in the absence of a positive family history. It is worth noting that although dozens of mutations have been identified in HDLS, the occurrence of de novo mutations was very rare [7, 12, 13]. Here, we confirmed a de novo c.1754-10 T > A mutation in a proband with no family history of HDLS, as confirmed by paternity tests. This finding also constitutes the first de novo splicing mutation ever reported in this disease.

CSF1R is a cell surface receptor for the CSF-1 factor and contains five immunoglobulin-like, extracellular ligand-binding domains; one transmembrane domain; and one tyrosine kinase intracellular domain [29]. CSF1R plays significant roles in regulating proliferation, survival, and differentiation of mononuclear phagocytic cells [30]. In the CNS, CSF1R is particularly highly expressed in microglial cells [31]. Most of the pathogenic mutations are located in the tyrosine kinase domain (encoded by exons 12 to 21 [32]) and can abrogate the CSF1R kinase activity, which affects the phosphorylation of downstream targets [13]. Of note, the mutation reported here is located at intron 12 and thus within the tyrosine kinase domain. Similar to our results, Xiaodong Yang et al. [21] reported a splicing mutation (c.1858 + 1G > T) in intron 13, which led to exon 13 skipping. In that study, the proband manifested progressive memory loss, slurred speech, limb stiffness, and weight loss, which are different from the symptoms reported in the present study. This difference highlights the significant clinical and genetic heterogeneities in HDLS [7]. Even so, we guess cognitive impairment and other clinical symptoms will appear a few months or years later because extensive white matter was involved in this patient. The prognosis of the patient is not optimistic as the abnormality progressed rapidly and there is no specific treatment for HDLS.

## Conclusion

In conclusion, we reported detailed clinical imaging features of an HDLS patient with no family history of this disease. In addition, we identified a de novo c.1754-10 T > A mutation in intron 12, which resulted in the skipping of exon 13 from the CSF1R mRNA. We also suggested that this skipping may further affect the normal function of the protein. This research indicated that HDLS should be taken into consideration even in the absence of family history. Persistent DWI hyperintense signals and genetic test are necessary to perform to differentiate HDLS from other diseases. Our study brings attention to the existence of a significant clinical heterogeneity in HDLS patients and contributes to the genetic understanding of this disease. Several limitations of this study must be addressed. First, the pathological examination of the proband’s brain tissue was unable to perform. Second, the specific function of CSF1R mutations was still unclear. We will perform functional experiments to elucidate the pathogenesis of CSF1R mutations in our future work.

## Data Availability

All data generated or analyzed during this study are included in this published article.
